# Supplementary material for: Observation of solid-state bidirectional thermal conductivity switching in antiferroelectric lead zirconate (PbZrO3)
Source: Nat Commun. 2022 Mar 23;13:1573. doi: 10.1038/s41467-022-29023-y (PMC8943065; doi:10.1038/s41467-022-29023-y)
Supplement: Supplementary file 1 — Supplementary Information [file 41467_2022_29023_MOESM1_ESM.pdf]

Observation of solid-state bidirectional thermal conductivity switching in antiferroelectric lead zirconate ($PbZrO_3$)

Kiumars Aryana[1], John A. Tomko[1], Ran Gao[2], Eric R. Hoglund[3], Takanori Mimura[3], Sara Makarem[3], Alejandro Salanova[3], Md Shafkat Bin Hoque[1], Thomas W. Pfeifer[1], David H. Olson[1], Jeffrey L. Braun[1], Joyeeta Nag[4], John C. Read[4], James M. Howe[3], Elizabeth J. Opila[1,3], Lane W. Martin[2,6], Jon F. Ihlefeld[3,5,†], and Patrick E. Hopkins[1,3,7,*]

[1]Department of Mechanical and Aerospace Engineering, University of Virginia, Charlottesville, Virginia 22904, USA
[2]Department of Materials Science and Engineering, University of California, Berkeley, Berkeley, CA 94720, USA
[3]Department of Materials Science and Engineering, University of Virginia, Charlottesville, Virginia 22904, USA
[4]Western Digital Corporation, San Jose, CA 95119, USA
[5]Department of Electrical and Computer Engineering, University of Virginia, Charlottesville, VA, 22904, USA
[6]Materials Sciences Division, Lawrence Berkeley National Laboratory, Berkeley, CA 94720, USA
[7]Department of Physics, University of Virginia, Charlottesville, Virginia 22904, USA
[†]jfi4n@virginia.edu
[*]phopkins@virginia.edu

(Supplementary Information)

Supplementary Note 1

In order to compare our experimental results for thermal conductivity of PZO as function of electric field against theoretical models, we turned to a Callaway-type model[1] that is widely used to estimate the thermal conductivity of crystalline materials[2–7]. This model takes various scattering parameters into account such as Umklapp, defect, and boundary scattering:

$$\kappa = \frac{k_B}{2\pi^2 v}\int_{k_B\Theta_D/\hbar}^{0} \tau \frac{\hbar^2\omega^2}{k_B^2T^2}\frac{e^{\hbar\omega/k_BT}}{(e^{\hbar\omega/k_B}-1)^2}\omega^2 d\omega, \tag{1}$$

where k_B and $\hbar$ are the Boltzmann's and Planck's constants, respectively, v is the speed of sound, Θ_D is the Debye temperature, τ is relaxation time, ω is the vibrational modes frequency, and T is temperature. We can estimate the relaxation time from different scattering mechanism via Matthiessen's rule:

$$\tau^{-1} = A\omega^4 + B\omega^2 Te^{-C/T} + v/d. \tag{2}$$

where d is the scattering length scale, and A, B, and C, are the scattering coefficients for impurity scattering ($A\omega^4$) and Umklapp scattering ($B\omega^2$ Texp$^{(-C/T)}$). Pertinent to this study, since the domain wall density increases upon electrical biasing, we change the scattering length scale (d) to match our thermal conductivity measurements. For this, we fix scattering length scale to the domain size in PZO and fit A, B, and C to our experimental data as a function of temperature as shown in Supplementary Fig. S1. According to our previous work[8], the antiferroelectric domains in our PZO film are oriented at 90°and display correlation lengths on the order of 3 and 30 nm for the [$\bar{1}$01] and [101] orientations, respectively. Assuming d = 3 and 30 nm, we fit for A, B, and C. Once, these scattering coefficients are determined, we calculate how much the scattering length scale, d, must change upon electrical biasing to match our experimental observations. According to these calculations, in order to reduce the thermal conductivity of PZO by ∼10% for 3 and 30 nm domain size, d must change by 18% and 20%, respectively. This degree of change in scattering length scale is comparable to previously reported values (10%) regarding percentage of change in the domain wall density for ferroelectric materials[9].

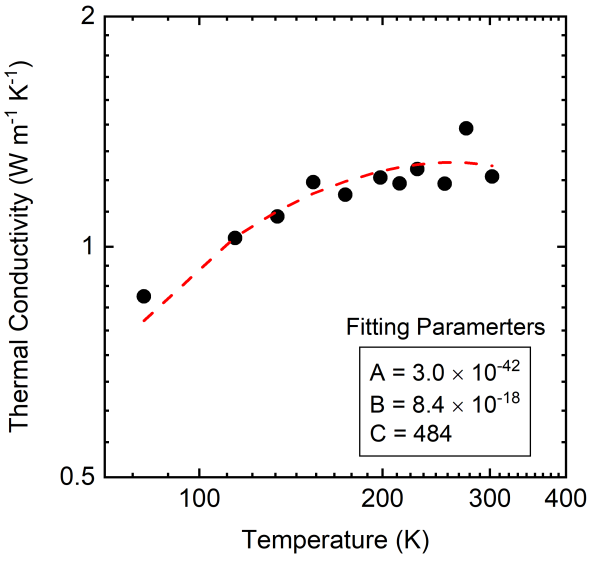

Figure S1. Measured thermal conductivity of PZO as a function of temperature. The dashed line shows the Callaway-type model's fit for the domain size of 3 nm.

Supplementary Note 2

Scanning transmission electron microscopy (STEM). Supplementary Fig. S2 shows images and selected-area electron diffraction patterns (SAED) before and after the *Pbam* to $Pm\bar{3}m$ phase transformation in the epitaxial film. The annular bright-field STEM images show that little to no microstructural or thickness changes occurred. Low-magnification annular bright-field is insensitive to the atomic scale displacements of Zr and O that occur during the phase transformation. We acquired SAED patterns to clearly show the phase transformation occurred, as shown in Supplementary Fig. S2(b,d). The fundamental Bragg reflections from the pseudo-cubic structure are circled with blue arrows and the ordered Bragg reflection that result from octahedral rotation are annotated with green and purple circles. The ordered reflections of the *Pbam* diffraction pattern are at $1/4\{hk0\}_{pc}$, which is consistent with the quadrupling of the unit-cell along $<110>_{pc}$ directions from both out-of-phase octahedral tilts and anti-parallel displacements. The selected-area aperture is larger than the 60 nm PZO layer so some of the Pt TDTR transducer is included in the diffracting region. The unmarked Bragg peaks in Supplementary Fig. S2(b,d) are from the Pt layer. Concentrating on the PZO Bragg peaks, there is a clear disappearance of the ordered Bragg reflections that flank the fundamental Bragg reflections upon heating above the Curie point, thus confirming the predicted phase transformation did indeed occur.

$<110>_{pc}$ zone axis selected-area diffraction patterns from the CSD film are shown in Supplementary Fig. S3 before (a) and after (b) heating. At room temperature the diffraction patterns have ordered reflections indicated by arrows, while after heating the ordered reflections disappeared. The disappearance of ordered reflections indicates the *Pbam* to $Pm\bar{3}m$ phase transformation occurred, much like in the epitaxial film. The same disappearance of ordered reflection is shown in lower index diffraction patterns in Fig. S4. Images in Supplementary Fig. S4 also show that minimal changes occurred in the microstructure. Some subtle changes did however occur. For example, the light and dark area marked my blue and red aperture position in Supplementary Fig. S4 are from different domain orientations, as shown by panels (b) and (c). Upon heating, the contrast became much more uniform and consisted of a single simple cubic structure, as shown in panel (d), consistent with the AFE-to-PE phase transformation.

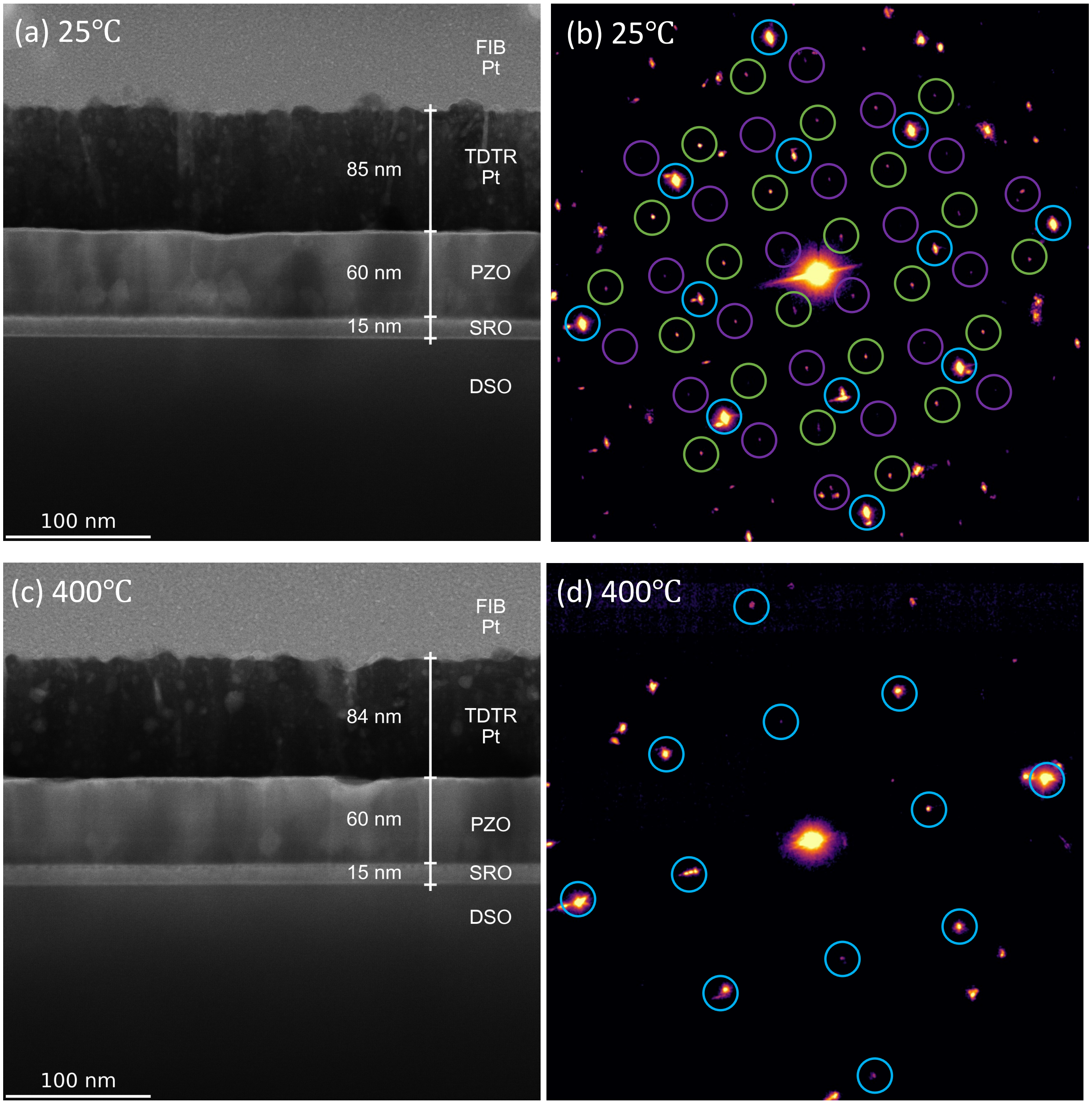

Figure S2. Annular bright-field STEM images of PZO at room temperature, 25°C, antiferroelectric and elevated temperature, 400°C, paraelectric phase and the corresponding selected area diffraction patterns. The annular bright-field STEM images show that the size of the thickness and grain size in the PZO film remains unaffected by heating. In the selected area diffraction patterns blue circles indicate Bragg reflections inherent to the pseudo-cubic perovskite unit-cell and the purple and green circles indicate $\frac{1}{4}\{hk0\}_{pc}$ ordered reflections from two domains that occur from periodic anit-parallel Ti displacements quadrupling the unit cell along $<110>_{pc}$. The lack of ordered reflections at 400°C shows that the PZO film has undergone a phase transition from orthorhombic space group *Pbam* to cubic $Pm\bar{3}m$. Bragg reflections that are not marked are from the Pt transducer.

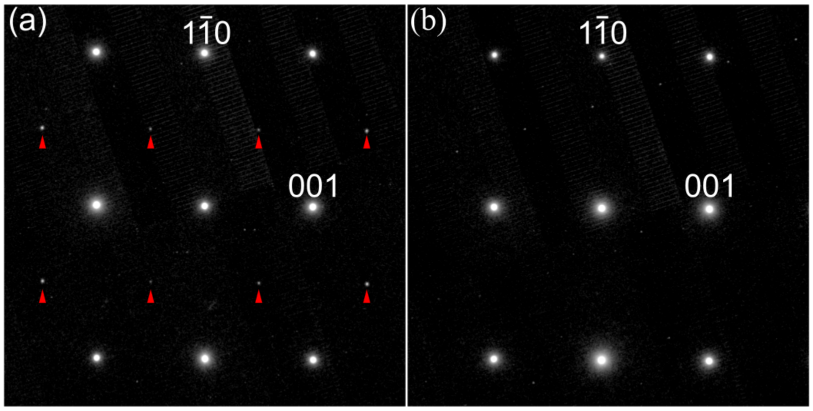

Figure S3. SAED of $\langle 110 \rangle_{pc}$ zone axis grains at (a) 25°C and (b) and 380°C. At 25°C $\frac{1}{2}$ $\langle 111 \rangle_{pc}$ ordered reflections are marked with red arrow and indicate the presence of the orthorhombic anti-ferroelectric phase. After heating no ordered reflections were observed in any $\langle 110 \rangle_{pc}$ zone axis, as seen in (b).

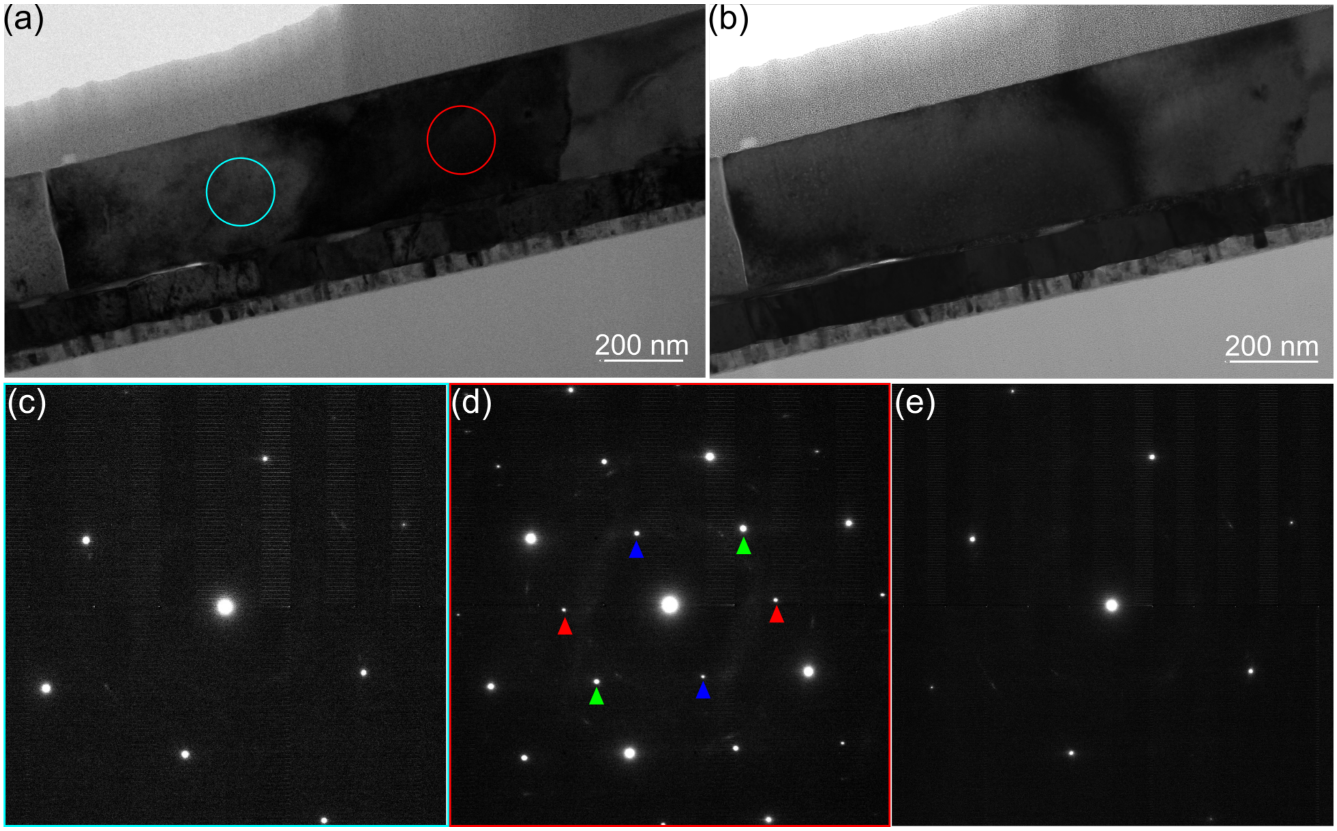

Figure S4. Bright-field image of a grain containing two [135] Zone axis at 25°C (a) with aperture position marked for the SAED patterns in (b,c). The SAED pattern in (b) did not have superlattice reflections. The SADP in (c) exhibited superlattice reflections $\frac{1}{3}$ $(\bar{3}\,\bar{4}3)_{pc}$, $\frac{1}{3}$ $(\bar{3}10)_{pc}$, and $\frac{1}{3}$ $(05\bar{3})_{pc}$ marked by red, blue, and green arrows, respectively. Bright-field image of the grain after heating to 285°C is shown in (d) with the respective SAED pattern shown in (e). One single grain existed and the SAED pattern did not contain superlattice reflections.

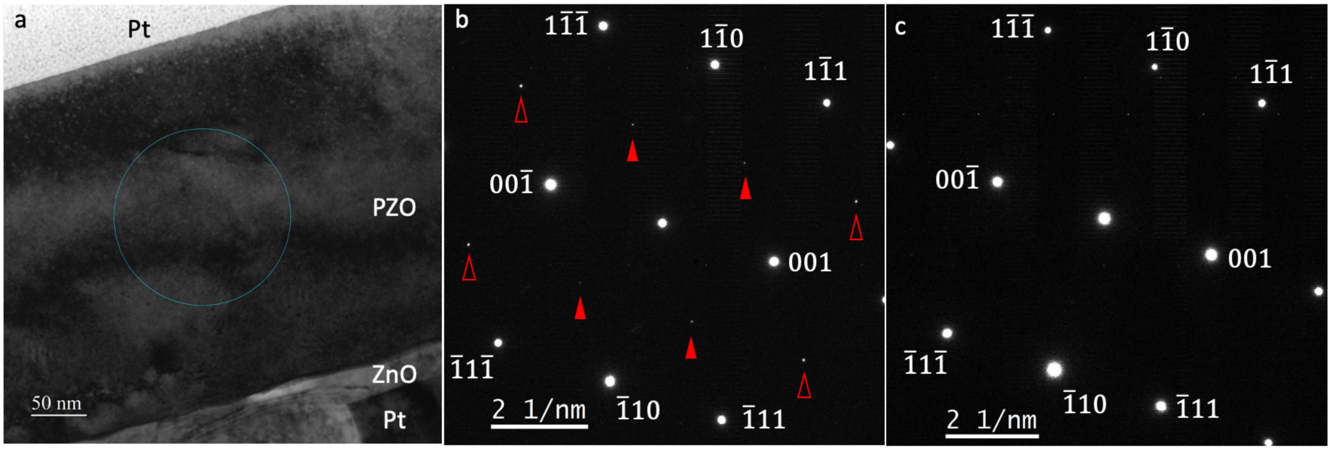

Figure S5. (a) Bright-field image of a <110>$_{pc}$ grain at 25 °C with the selected-area aperture position for the diffraction pattern in (b) circled. The SADP exhibits ordered reflections (red arrows) at $\frac{1}{2}$ <111>$_{pc}$, characteristic of an ordered octahedral arrangement in the orthorhombic anti-ferroelectric phase. Slightly brighter $\frac{1}{2}$ <113>$_{pc}$ ordered reflections are also indicated by hollow red arrows. (c) The ordered reflections disappear upon heating to 380 °C, indicating a transition to the cubic ferroelectric $PbZrO_3$ phase as reported in similar studies[10,11].

Supplementary Note 3

Hot disk measurement. The temperature-dependent thermal conductivity of a pair of commercial bulk PZO samples were measured using a hot disk (TPS 3500, Thermtest) based on the transient plane source technique[12,13] and the results are presented in supplementary Fig. S6. A Kapton sensor of 3.189 mm radius was utilized for this purpose. Prior to the PZO measurements, the hot disk was calibrated against two reference standards (stainless-steel and BK7 window glass) at room temperature. The measured values of the two calibrations were 14 ± 7 and 1.0 ± 0.1 W m^{-1} K^{-1}, respectively, in good agreement with literature[14–16]. As the density of the bulk PZO samples (6.93 g cm^{-3}) was lower than the literature reported value (7.98 g cm^{-3},[17]), the measured thermal conductivities were corrected for porosity effects[18].

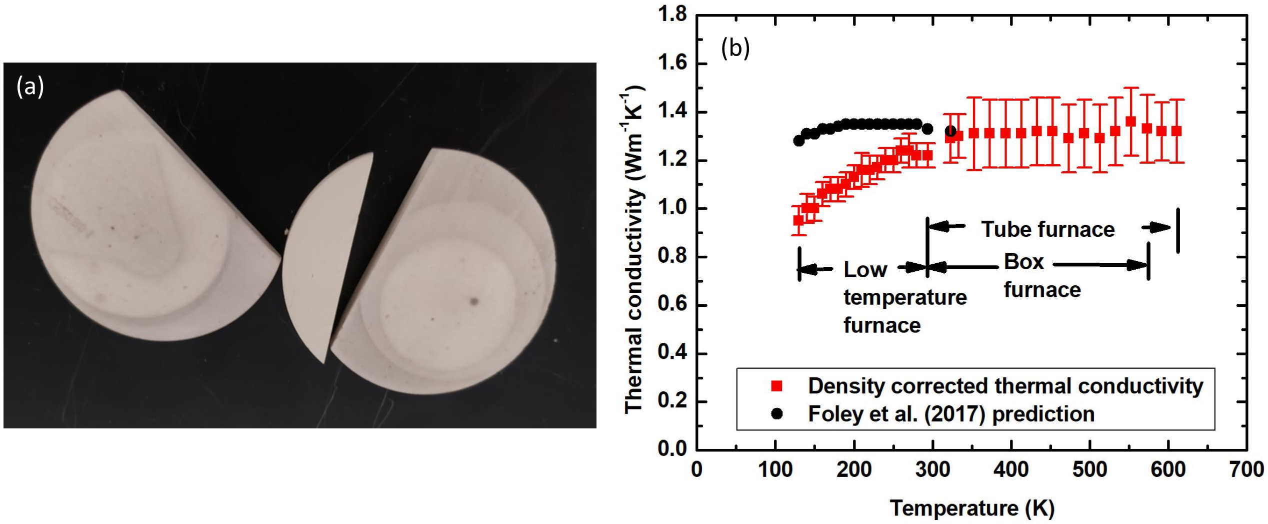

Figure S6. (a) The bulk commercial PZO sample used for hot disk measurement. (b) Thermal conductivity as a function of temperature for bulk PZO (red squares). The uncertainty of the thermal conductivity values incorporates the standard deviation among multiple measurements, uncertainty associated with the PZO heat capacity, and porosity correction factor.

With regards to the disagreement in the measured thermal conductivity between the bulk and thin film samples, we should mention that we contacted several vendors to purchase high-quality PZO in bulk and we found it impossible to find a company that makes 100% high quality bulk PZO with no porosity and defects. Nonetheless, to investigate our hypothesis regarding the existence of porosity and impurities in our bulk PZO, we performed SEM and EDS measurements and found that not only a significant degree of porosity exists in our measured bulk PZO as depicted in Supplementary Fig. S7 but also, we found signatures of Ti impurities and non-stoichiometry. Pure $PbZrO_3$ should be 20 mole percent Pb, but this ceramic was closer to 19 mole% as presented in Supplementary Table S1. On the other hand, the PZO films made for this study are epitaxially grown and the balance of Pb vaporization and Pb flux during growth results in improved stoichiometry films. Furthermore, the epitaxial films have a very high degree of crystallinity and crystal perfection. In the light of these evidences, it is impossible to compare our thermal conductivity results for thin films with that of the bulk ceramics to which we have access.

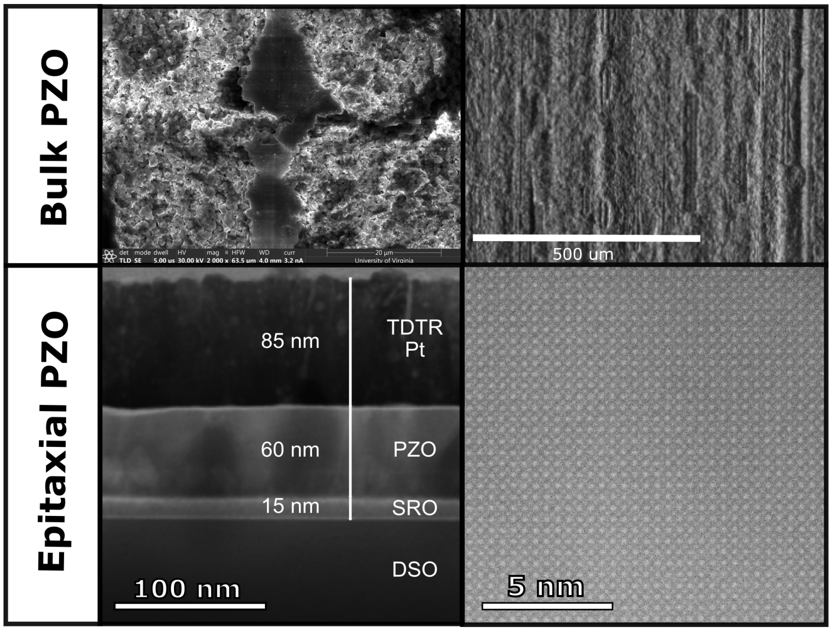

Figure S7. The PZO micro- and nanostructure for bulk and thin film.

Table S1. Electron dispersive spectroscopy (EDS) performed in scanning electron microscope (SEM) showing compositions in the bulk PZO sample.

Spectrum 1							
**Element**	**Line Type**	**Apparent Concentration**	**k Ratio**	**Wt%**	**Wt% Sigma**	**At.%**	**Standard Label**
**O**	K series	6.68	0.02248	14.64	0.14	61.4	SiO2
**Ti**	K series	0.17	0.00172	0.24	0.02	0.3	Ti
**Zr**	L series	23.73	0.23731	26.06	0.11	19.2	Zr
**Pb**	L series	60.03	0.57898	59.05	0.14	19.1	PbTe
**Total:**				100.00			

Supplementary Note 4

In order to calculate the steady-state temperature rise due to laser heating, we use a model developed in our previous work[19]. The temperature rise in this case largely depends on the absorption coefficient of the $SrRuO_3$ at wavelength of 532 nm, the beam size (12 um), and the delivered power. According to the literature, for 100 nm $SrRuO_3$ the absorption is near 80%[20]. Although the absorption of $SrRuO_3$ depends on the deposition process and the quality of the film, we take 80% as the upper limit for the absorption of $SrRuO_3$. Furthermore, the measured power is before the laser passes a few mirrors and the objective. Assuming 10% power drop from the measured point to the surface of the sample, the estimated temperature rises within the probed region for the 20, 40, and 60 mW is approximately 135, 271, and 406 K. This agrees with the observed trend in Fig. 5(b) in the main manuscript. Since the beam profile is Gaussian, there is a Gaussian temperature rise on the surface of the sample.

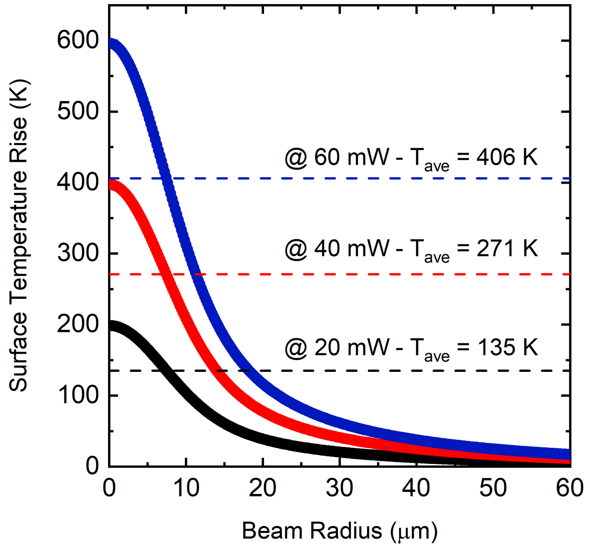

Figure S8. Temperature rise profile as a function of probe beam radius due to a Gaussian CW heater beam at different powers.

Supplementary Note 5

Substrate effect on PZO thermal conductivity measurements. In this section we discuss the effect of substrate on our thermal conductivity measurement of PZO thin film. Changes in temperature can result in changes in the thermal conductivity of substrate and create fictitious thermal conductivity increase in our measurements. We investigate this by separately measuring the thermal conductivity of DSO substrate as a function of temperature. Supplementary Fig. S9a shows the experimental data with their corresponding theoretical fit at room and elevated temperatures. As depicted in Supplementary Fig. S9b, the thermal conductivity of DSO substrate decreases at higher temperatures which is in great agreement with previous studies[21]. This indicates that, if the measured thermal conductivity of PZO was affected by the substrate, we should have observed a reduction in the thermal conductivity which is contrary to our experimental observation. Unlike DSO, the thermal conductivity of PZO increases with temperature and the fact that we see this increase is an indicative of the independence of our measurements from that of the substrate.

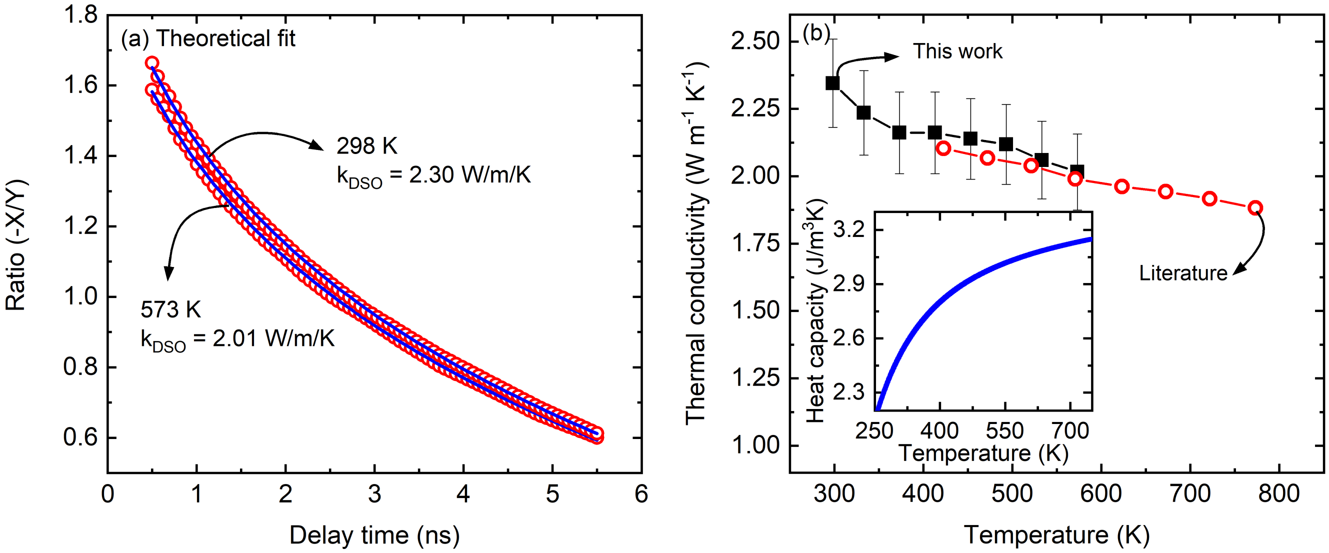

Figure S9. (a) Experimental data and the theoretical fit for the thermal conductivity measurements at room temperature and 573 K. (b) Thermal conductivity of DSO substrate as a function of temperature. The uncertainty is calculated based on 10% variations in the transducer thickness. The inset shows the heat capacity of DSO estimated using equation provided in Ref.[21]

.

References

1. Callaway, J. Model for lattice thermal conductivity at low temperatures. *Phys. Rev.* **113**, 1046 (1959).
2. Thacher, P. D. Effect of boundaries and isotopes on the thermal conductivity of lif. *Phys. Rev.* **156**, 975 (1967).
3. Wei, L., Kuo, P., Thomas, R., Anthony, T. & Banholzer, W. Thermal conductivity of isotopically modified single crystal diamond. *Phys. review letters* **70**, 3764 (1993).
4. Olson, J. *et al.* Thermal conductivity of diamond between 170 and 1200 k and the isotope effect. *Phys. Rev. B* **47**, 14850 (1993).
5. Mingo, N., Yang, L., Li, D. & Majumdar, A. Predicting the thermal conductivity of si and ge nanowires. *Nano Lett.* **3**, 1713–1716 (2003).
6. Foley, B. M. *et al.* Phonon scattering mechanisms dictating the thermal conductivity of lead zirconate titanate (pbzr1- x ti x o3) thin films across the compositional phase diagram. *J. Appl. Phys.* **121**, 205104 (2017).
7. Scott, E. A. *et al.* Reductions in the thermal conductivity of irradiated silicon governed by displacement damage. *Phys. Rev. B* **104**, 134306 (2021).
8. Gao, R. *et al.* Ferroelectricity in $Pb_1 + \delta ZrO_3$ thin films. *Chem. Mater.* **29**, 6544–6551 (2017).
9. Ihlefeld, J. F. *et al.* Room-temperature voltage tunable phonon thermal conductivity via reconfigurable interfaces in ferroelectric thin films. *Nano Lett.* **15**, 1791–1795 (2015).
10. Viehland, D. Transmission electron microscopy study of high-Zr-content lead zirconate titanate. *Phys. Rev. B* **52**, 778 (1995).
11. Corker, D., Glazer, A., Whatmore, R., Stallard, A. & Fauth, F. A neutron diffraction investigation into the rhombohedral phases of the perovskite series. *J. Physics: Condens. Matter* **10**, 6251 (1998).
12. Gustafsson, S. E. Transient plane source techniques for thermal conductivity and thermal diffusivity measurements of solid materials. *Rev. Sci. Instruments* **62**, 797–804 (1991).
13. Gustavsson, M., Karawacki, E. & Gustafsson, S. E. Thermal conductivity, thermal diffusivity, and specific heat of thin samples from transient measurements with hot disk sensors. *Rev. Sci. Instruments* **65**, 3856–3859 (1994).
14. Ho, C. Y. & Chu, T. Electrical resistivity and thermal conductivity of nine selected AISI stainless steels. Tech. Rep., Thermophysical and Electronic Properties Information Analysis Center ... (1977).
15. Assael, M., Botsios, S., Gialou, K. & Metaxa, I. Thermal conductivity of polymethyl methacrylate (PMMA) and borosilicate crown glass BK7. *Int. J. Thermophys.* **26**, 1595–1605 (2005).
16. Braun, J. L., Olson, D. H., Gaskins, J. T. & Hopkins, P. E. A steady-state thermoreflectance method to measure thermal conductivity. *Rev. Sci. Instruments* **90**, 024905 (2019).
17. Ko, J., Roleder, K. & Bussmann-Holder, A. Determination of elastic stiffness coefficients of lead zirconate single crystals in the cubic phase by Brillouin light scattering. In *IOP Conference Series: Materials Science and Engineering*, vol. 54, 012002 (IOP Publishing, 2014).
18. Yoshida, I. Thermal conduction in ferroelectric ceramics. *J. Phys. Soc. Jpn.* **15**, 2211–2219 (1960).
19. Braun, J. L., Szwejkowski, C. J., Giri, A. & Hopkins, P. E. On the steady-state temperature rise during laser heating of multilayer thin films in optical pump–probe techniques. *J. Heat Transf.* **140** (2018).
20. Lee, S., Apgar, B. A. & Martin, L. W. Strong visible-light absorption and hot-carrier injection in tio_2/$srruo_3$ heterostructures. *Adv. Energy Mater.* **3**, 1084–1090 (2013).
21. Hidde, J., Guguschev, C., Ganschow, S. & Klimm, D. Thermal conductivity of rare-earth scandates in comparison to other oxidic substrate crystals. *J. Alloy. Compd.* **738**, 415–421 (2018).
