# Supplementary material for: Observation of solid-state bidirectional thermal conductivity switching in antiferroelectric lead zirconate (PbZrO3)
Source: Nat Commun. 2022 Mar 23;13:1573. doi: 10.1038/s41467-022-29023-y (PMC8943065; doi:10.1038/s41467-022-29023-y)
Supplement: Supplementary file 4 — Lasing Reporting Summary [file 41467_2022_29023_MOESM4_ESM.pdf]

nature research

Corresponding author(s): Patrick E. Hopkins

Lasing Reporting Summary

Nature Research wishes to improve the reproducibility of the work that we publish. This form is intended for publication with all accepted papers reporting claims of lasing and provides structure for consistency and transparency in reporting. Some list items might not apply to an individual manuscript, but all fields must be completed for clarity.

For further information on Nature Research policies, including our data availability policy, see Authors & Referees.

Experimental design

Please check: are the following details reported in the manuscript?

1. Threshold

Item	Yes	No	Response
Plots of device output power versus pump power over a wide range of values indicating a clear threshold	☐	☒	not relevant

2. Linewidth narrowing

Item	Yes	No	Response
Plots of spectral power density for the emission at pump powers below, around, and above the lasing threshold, indicating a clear linewidth narrowing at threshold	☐	☒	not relevant
Resolution of the spectrometer used to make spectral measurements	☐	☒	not relevant

3. Coherent emission

Item	Yes	No	Response
Measurements of the coherence and/or polarization of the emission	☐	☒	not relevant

4. Beam spatial profile

Item	Yes	No	Response
Image and/or measurement of the spatial shape and profile of the emission, showing a well-defined beam above threshold	☒	☐	Methods section

5. Operating conditions

Item	Yes	No	Response
Description of the laser and pumping conditions *Continuous-wave, pulsed, temperature of operation*	☒	☐	Methods section
Threshold values provided as density values (e.g. W cm^{-2} or J cm^{-2}) taking into account the area of the device	☐	☒	not relevant

6. Alternative explanations

Item	Yes	No	Response
Reasoning as to why alternative explanations have been ruled out as responsible for the emission characteristics *e.g. amplified spontaneous, directional scattering; modification of fluorescence spectrum by the cavity*	☐	☒	not relevant

7. Theoretical analysis

Item	Yes	No	Response
Theoretical analysis that ensures that the experimental values measured are realistic and reasonable *e.g. laser threshold, linewidth, cavity gain-loss, efficiency*	☐	☒	not relevant

8. Statistics

Item	Yes	No	Response
Number of devices fabricated and tested	☐	☒	not relevant
Statistical analysis of the device performance and lifetime (time to failure)	☐	☒	not relevant
